# Spatial Distribution Characteristics of Healthcare Facilities in Nanjing: Network Point Pattern Analysis and Correlation Analysis

**DOI:** 10.3390/ijerph13080833

**Published:** 2016-08-18

**Authors:** Jianhua Ni, Tianlu Qian, Changbai Xi, Yikang Rui, Jiechen Wang

**Affiliations:** 1 Department of Geographic Information Science, Nanjing University, Nanjing 210093, China; neejianhua@126.com (J.N.); qtl1234@126.com (T.Q.); xicb11@gmail.com (C.X.); rui_yikang@outlook.com (Y.R.); 2 Department of Resources Environment and Tourism Management, West Anhui University, Luan 237012, China; 3 Jiangsu Provincial Key Laboratory of Geographic Information Science and Technology, Nanjing 210093, China; 4 Jiangsu Center for Collaborative Innovation in Geographical Information Resource Development and Application, Nanjing 210023, China

**Keywords:** healthcare facilities, network kernel density estimation, network K-function, street centrality, correlation analysis

## Abstract

The spatial distribution of urban service facilities is largely constrained by the road network. In this study, network point pattern analysis and correlation analysis were used to analyze the relationship between road network and healthcare facility distribution. The weighted network kernel density estimation method proposed in this study identifies significant differences between the outside and inside areas of the Ming city wall. The results of network K-function analysis show that private hospitals are more evenly distributed than public hospitals, and pharmacy stores tend to cluster around hospitals along the road network. After computing the correlation analysis between different categorized hospitals and street centrality, we find that the distribution of these hospitals correlates highly with the street centralities, and that the correlations are higher with private and small hospitals than with public and large hospitals. The comprehensive analysis results could help examine the reasonability of existing urban healthcare facility distribution and optimize the location of new healthcare facilities.

## 1. Introduction

The Chinese economy has been growing at a spectacular rate and people’s living standards have improved significantly since the reform and opening up. At the same time, Chinese healthcare resources have developed significantly to cater for rising demand. Average bed numbers per 1000 people have grown from 0.15 in 1949 to 4.55 in 2013 [1]. However, imbalanced distribution of health resources has led to severe inequality between cities and rural areas, which largely influences social stability and harmony in China. According to the Chinese government, about 80 percent of health resources (e.g., hospitals, bed numbers, and practitioners) are allocated in Chinese cities [2]. In addition, the size of large hospitals has been expanding excessively and the majority of health resources are concentrated in large city hospitals [3]. For the rapid expansion of large Chinese cities, there is an urgent need to study spatial organization and distribution of patterns of healthcare facilities to optimize their location selection and spatial allocation [4].

Spatial pattern analysis has been examined widely to explore global or local spatial distribution patterns of urban activities. It can be classified into first-order and second-order effects of a spatial process [5,6]. Kernel density estimation (KDE) and Ripley’s K-function are two of the most popular methods for analyzing the first-order and second-order properties of a point event distribution [6,7]. KDE has been used to analyze “hot spots” of point events, such as the traffic hazard intensity of bicycles [8], wildlife–vehicle accident analysis [9], and road accident hot-spot analysis and classification [10]. Ripley’s K-function has been used to test whether any pair of events is spatially dependent or uniform by distance measure. In fact, these studies are based on the assumption of infinitely continuous planar space in which distances are measured as a straight-line (Euclidean) distance.

However, many kinds of point events associated with urban activities are constrained by road networks in the real world. Those events can be classified into on-network events and alongside-networks events. Almost all facilities in urbanized areas are regarded as alongside-network events. The use of a planar point pattern analysis (PPA) method over a Euclidean space has limitations for analyzing these events because they are often constrained to only the network portion of the 2-D Euclidean space, the so-called network space [11]. Therefore, the traditional planar PPA methods lead to false analysis results for the network-constrained point events. Borruso and Giuseppe [12,13] introduced network density estimation (NDE) to analyze patterns of bank and insurance branch location based on network distances. Xie and Yan [14,15] developed a network KDE (NetKDE) approach to characterize the spatial patterns of traffic accidents on roadways over a network space. Okabe et al. [16] showed that previous kernel methods may yield biased conclusions and developed two unbiased kernel functions on a network for the density estimation of traffic accidents. Okabe and Yamada [17] first proposed the concept of network K-function for analyzing the distribution of points on a network. Yamada and Thill [18] proposed that the planar K-function analysis method over-detected clustered patterns of point events and demonstrated the benefits of using a network space for traffic accident data.

Urban road network plays a very important role in shaping the formation of urban activities. Street centrality is one of the most powerful determinants for urban planners and designers to understand how a city works and to decide where renovation and redevelopment need to be placed [19,20]. Many studies have attempted to investigate the spatial relationship between urban activities and road network by using their correlation results. Porta et al. [19,20] examined correlations of economic activities with the urban road network. They found that the location of economic activities is highly correlated with the road network. In addition, Wang et al. [21] found a high correlation between population density distribution and road network. Rui and Ban [22] further explored the relationship between different street centralities and land-use types in Stockholm.

Network point pattern and correlation analysis have been used widely to study the relationship between road network and facility distribution. However, despite its relative popularity, few studies have focused on the macroscopic distribution pattern of healthcare facilities using both of these methods. Furthermore, a major limitation has been identified when using traditional NetKDE methods. They probably calculate misleading hot spots because they are independent with non-spatial factors of point events [23]. Even if the comprehensive strength of one facility point is thousands of times that of another point event, both have the same influential intensity if they are at the same location. As we know, a larger hospital has a greater ability to accommodate more patients. Therefore, in this study, we proposed a weighted network KDE method based on a crucial non-spatial attribute of point events to address this problem. Due to competition or cooperation, there may be significant relationships between pairs of hospitals, and between hospitals and pharmacies on a distance measure. Network auto K-function and cross K-function were used to explore the spatial cluster pattern analysis of hospitals and interrelationships between hospitals and pharmacy stores along the network. To examine spatial distribution differences of different kinds of hospitals along the network, the correlation analysis between different categorized hospitals and street centrality was investigated for further comparable analysis of the results.

## 2. Study Area and Data Sources

### 2.1. Study Area

**Figure 1:**
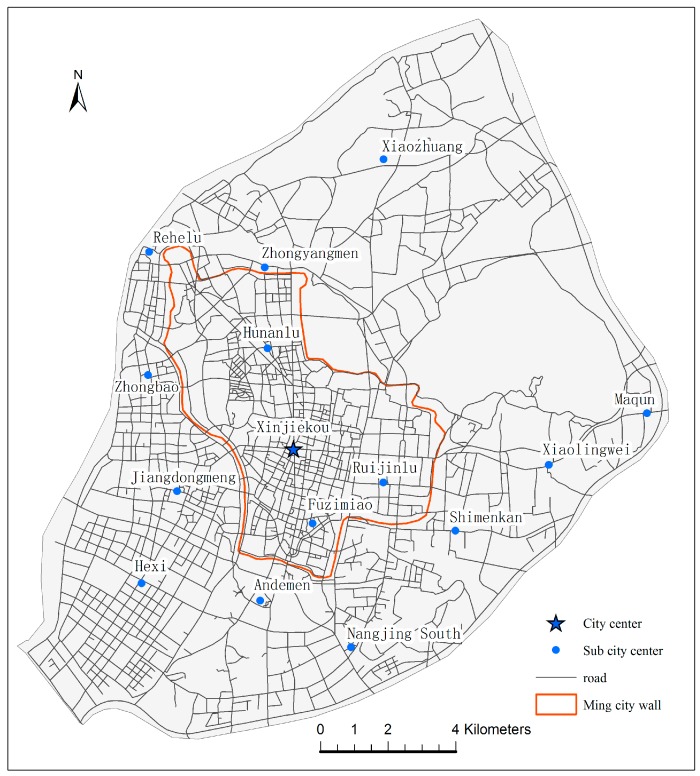
City centers with road network in study area.

Nanjing is the capital of Jiangsu province, which is located slightly inland from the coast of China. Nanjing has long been one of China’s most important cities. With an urban population of 6.43 million in 2013 [24], Nanjing is the second largest city in the East China region, after Shanghai. The study area is located in the main urban districts of Nanjing, which are surrounded by the beltway and Yangtze River. The downtown area is defined as the area inside the 600-year-old Ming city wall of the main urban districts (red color in Figure 1). There are 15 city centers within the main urban districts. Figure 1 shows the locations of one city center and 14 sub-centers with roads representing 2801 edges. In the center of the main urban districts, Xinjiekou is the central business and commercial district of Nanjing.

### 2.2. Data Sources

**Figure 2:**
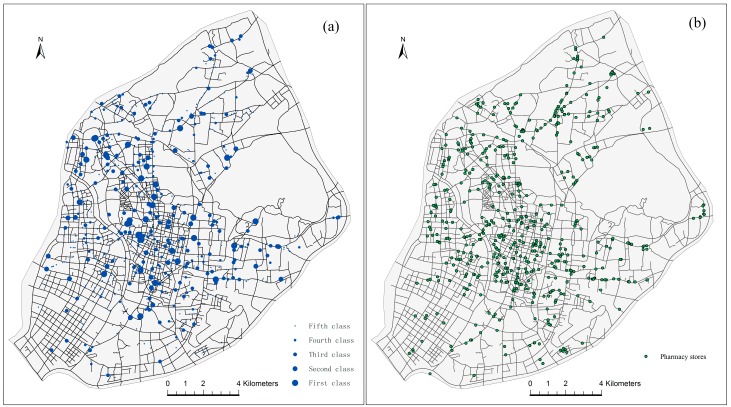
(**a**) Distribution of hospitals and (**b**) pharmacy stores in main urban districts.

For this study, the data of healthcare facilities include hospitals and pharmacy stores, which are all indicated by a point pattern in a geographic information system (GIS) environment, as shown in Figure 2. There are 732 hospital points with 33,366 bed numbers in total, according to the 2012 Jiangsu Provincial Commission of Health and Family Planning [25]. Figure 2a shows the spatial distribution of hospitals in the main urban districts of Nanjing. The blue circles of different sizes represent different comprehensive strength of the hospitals in these locations. As shown in Table 1, hospitals can be divided by ownership (private and public hospitals) and comprehensive strength (first class to fifth class) [26]. The first- and fifth-class hospitals are the strongest and weakest hospitals, respectively, in terms of comprehensive strength. First- and second-class hospitals are usually large hospitals belonging to the Chinese government. Third-class hospitals are community hospitals located in residential areas. Fourth- and fifth-class hospitals are usually small hospitals or clinics. There are 569 pharmacy stores within the main urban districts. Figure 2b shows the spatial distribution of pharmacy stores in main urban districts. Spatial data of the road networks are from the Nanjing Transportation Bureau.

## 3. Methods

### 3.1. Network Kernel Density Estimation

Network kernel density estimation is one of the most important network analysis methods for density measure, which estimates the density of point events on a network according to a kernel density function. Instead of a planar Euclidean distance measure in planar KDE, NetKDE uses the shortest-path distance along the network:

(1) $$\lambda \left(s\right)=\overset{n}{\underset{i=1}{\sum }}\frac{1}{r}k\left(\frac{d_{is}}{r}\right)$$

where λ(s) is the NetKDE density at location *s*, *i* represents the point event, *r* is the bandwidth of the NetKDE, and *k*() is a kernel function of the ratio of $d_{is}$ to *r* with the distance decay effect. A number of forms of model functions, known as kernel functions, can be used to measure the “distance decay effect” in the spatial weights *k*, such as Gaussian, Conic, Quartic, negative exponential, and Epanichnekov [14,27,28]. Here, we use the most commonly used Gaussian function:

(2) $$k\left(\frac{d_{is}}{r}\right)=\frac{1}{\sqrt{2\pi }}\times exp\left(-\frac{d_{is}^{2}}{2r^{2}}\right)\times B_{i},\;\text{when}\;0<d_{is}\leq r$$

(3) $$k\left(\frac{d_{is}}{r}\right)=0,\;\text{when}\;d_{is}>r$$

where $B_{i}$ is the non-spatial factor of the point event i, and the function form of (1) is the common Gaussian function if $B_{i}$ = 1. In this study, the parameter $B_{i}$ represents the bed numbers of the hospital in location *i*; in other words, the more bed numbers that a hospital has, the higher is the hospital’s density.

### 3.2. Network K-Function

The network K-function method is one of the most important network analysis methods for distance measure, which is used to test the hypothesis that points are uniformly and independently distributed over a network [17]. The network K-function method includes the network auto K-function method and the network cross K-function method. The main distinction is that the auto K-function method deals with a set of points of a single kind (e.g., retail stores) and considers the shortest-path network distances between these points. By contrast, the cross K-function method deals with two sets of points of different kinds (e.g., retail stores and roads) and instead considers the shortest-path network distances between these two different kinds of points. The network auto K-function K(t) at place $p_{i}$ is defined as follows:

(4) $$K\left(t\right)=\frac{1}{\rho }\frac{\sum_{i=1}^{n}n(t|p_{i})}{n}$$

where $n(t|p_{i})$ is the number of points that are within shortest-path distance t from point $p_{i}$. In addition, $\rho =\left(n-1\right)∕\left|\tilde{S}\right|$ is the density of points on the network (the numerator n−1 means $p_{i}$ is removed from point set *P*; $\left|\tilde{S}\right|$ is the length of a subnetwork of $\tilde{S}$). The Monte Carlo simulation (MCS) method is often used to test the distribution pattern of point events constrained by the road network. Whether these points are uniformly and independently distributed over a network depends on the differences between the observed K-function values and the completely spatial random (CSR) point pattern test. If the corresponding K-function values *K(l)* are in the range of the CSR, the point set *P* is in a random distribution. If *K(l)* is above the upper CSR bound, the point set *P* is in a cluster distribution. If *K(l)* is below the lower CSR bound, the point set *P* is in a dispersion distribution. In addition, the cross K-function uncovers the spatial interrelationship of two point sets. It studies whether the distribution of one point set influences the pattern of another point set. If the observed curve is above the upper envelope curve, one point set tends to cluster around another point set. However, if the observed curve is below the lower envelope curve, we can reject the CSR hypothesis with confidence. That is, one point set tends to be dispersed from another point set.

### 3.3. Multiple Centrality Assessment Model and Correlation Coefficient

Street centrality has been one of the most useful indexes to describe the accessibility of one facility or one place within a road network [29]. Exploring the relationship between street centrality and facility could help analyze the spatial distribution pattern of healthcare facilities. Three major centrality indexes usually exist: betweenness centrality ($C^{B}$), closeness centrality ($C^{C}$), and straightness centrality ($C^{S}$). Various centrality indexes represent road accessibility from different aspects. The *C^B^* measure indicates the extent to which a node is passed by the shortest path between pairs of other nodes in the network [30], such as:

(5) $$C_{i}^{B}=\frac{1}{\left(N-1\right)\left(N-2\right)}\overset{N}{\underset{j=1;\;k=1;\;j\neq k\neq i}{\sum }}\frac{n_{jk}\left(i\right)}{n_{jk}}$$

where $C_{i}^{B}$ is the betweenness of node *i*, *N* is the number of nodes, $n_{jk}$ is the number of shortest paths from node *j* to node *k*, while $n_{jk}\left(i\right)$ is the number of shortest paths through node *i*.

The $C^{C}$ measure illustrates how close a node is to all other surrounding nodes in the network, such as:

(6) $$C_{i}^{C}=\frac{N-1}{\sum_{j\neq 1,\;j\neq i}^{N}d_{ij}}$$

where $C_{i}^{C}$ is the closeness of node *i* and $d_{ij}$ is the shortest-path distance between nodes *i* and *j*.

The $C^{S}$ measure indicates the extent to which a node can be reached directly on the Euclidean distance from all other nodes, such as:

(7) $$C_{i}^{S}=\frac{1}{N-1}\overset{N}{\underset{j=1;\;j\neq i}{\sum }}\frac{d_{ij}^{Eu}}{d_{ij}}$$

where $C_{i}^{S}$ is the straightness of node *i*, $d_{ij}^{Eu}$ is the Euclidean distance between nodes *i* and *j*, and $d_{ij}$ is the shortest-path distance between them.

In this study, a simple linear correlation coefficient was computed to analyze the differences between different categories or ownership of hospitals and road network in the same raster framework. The linear correlation coefficient is Spearman’s *r*, ranging from −1 to 1, which was proposed by Charles Spearman as a measure of the strength of an association between two variables [31].

## 4. Results

### 4.1. Weighted NetKDE Analysis for Detecting Hot Spots

**Figure 3:**
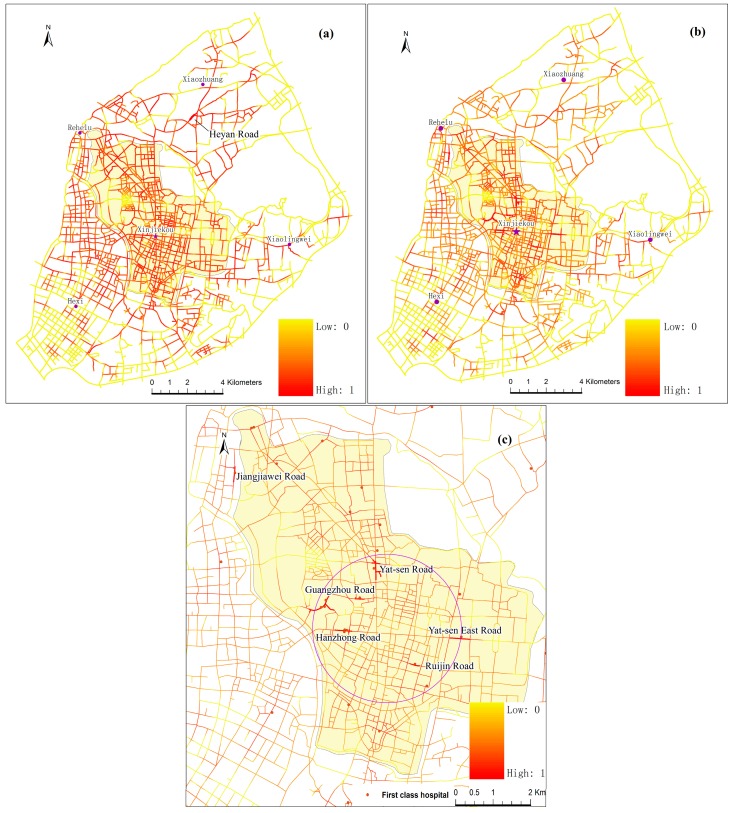
(**a**) Unweighted NetKDE results; (**b**) weighted NetKDE results; and (**c**) weighted NetKDE results around the city center for hospitals.

In the NetKDE analysis, few studies consider the influence of non-spatial factors of service facilities on the analysis. Bed numbers are considered the most crucial factor for hospitals in many research studies [32,33,34]. Hence, to identify more details about the density of hospital locations, hospital facilities were weighted by their bed numbers. The computation of NetKDE values was implemented by Microsoft Visual C++ (Microsoft, Redmond, WA, USA) and ESRI ArcObjects (ESRI, Redlands, CA, USA) programming languages. It is generally agreed that the choice of kernel functions do not affect the results significantly [35]. However, the choice of bandwidth (h) is always an important issue in KDE applications [14]. Porter and Reich [36] suggested that a 100–300 m bandwidth was suitable for the study of urban economic activities. However, in fact, there are not so many hospitals along the road network. Therefore, a 600 m bandwidth was chosen to calculate NetKDE of hospital facilities after numbers of experiments. The cell size for the output KDE raster dataset was set as 20 m. Figure 3a is the NetKDE result of all the hospitals in the main urban districts based on their bed numbers. For contrast, the result of unweighted hospitals’ NetKDE was also computed, as shown in Figure 3b.

Figure 3b,c show that the weighted NetKDE method identifies significant differences between the outsides and insides of the city wall. There are seven outstanding road segments with network density values of more than 0.53, and six of them are within the city wall, while the seventh road segment is just 240 m from the edge of the city wall. The higher NetKDE values are mainly located in the, Yat-sen East Road, Yat-sen Road, Jiangjiawei Road, Guangzhou Road, and Hanzhong Road, as shown by the red lines in Figure 3c. Three first-class hospitals are concentrated in the intersections of Guangzhou Road and Lhasa Road, where there are the highest NetKDE values, ranging from 0.72 to 1.0. Interestingly, the seven top hospitals (more than 1000 bed numbers) are all located within the circle of the city center—Xinjiekou—with a radius of about 5 km. Thus, our results show higher network density values within the city wall. However, most of the NetKDE values are lower than 0.3 outside the city wall.

However, the unweighted NetKDE method does not identify detailed intensity differences of the hospital locations along the road network. Figure 3a shows the highest NetKDE values are around the south area of Xiaozhuang sub-center, which is far away from the city center. The values range from 0.73 to 1.0. Although there are large numbers of hospitals in these areas, most are lower-class hospitals or small clinics with few bed numbers. Thus, our weighted analysis shows lower NetKDE values in these areas. However, in Guangzhou Road, around which are gathered a number of large and small hospitals, the NetKDE values range from 0.25 to 0.46. This is because the unweighted NetKDE method depends on the density of point events along networks. The values may be underestimated if there are few hospital points but with a large number of bed resources somewhere. By contrast, the values may be overestimated if there are many hospital points with few bed resources somewhere. By using the weighted NetKDE method, significant differences between the outside and inside areas of the Ming city wall are successfully identified, which is consistent with the fact that the majority of health resources is concentrated in city areas.

### 4.2. Spatial Cluster Pattern Analysis

**Figure 4:**
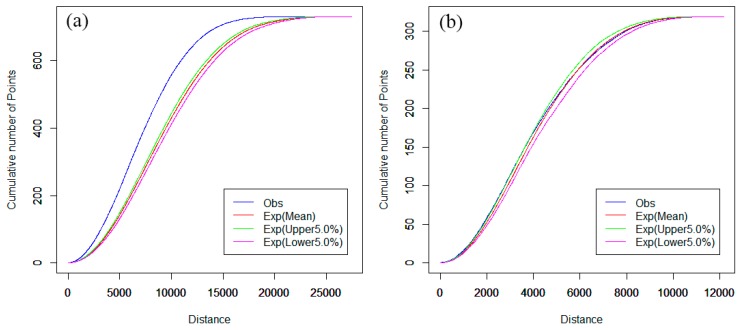
(**a**) Network auto K-function analysis of main urban districts and (**b**) network auto K-function analysis of downtown area.

The spatial cluster analysis of hospitals was compared for the downtown area and the main urban districts of Nanjing. The computation of network K-function values was implemented by SANET software (Tokyo University, Tokyo, Japan). The network auto K-function analysis results are shown in Figure 4, and are obtained from the R language window.

In Figure 4, the parameters “Exp(Lower 5.0%)” and “Exp(Upper 5.0%)” indicate the upper and lower envelope curves for the one-sided significance level is 5% [37]. The parameter “Exp(Mean)” indicates the expectation curve of random spatial distribution of point events. The parameter “Obs” indicates the observed K-function curve of point events. The horizontal axis indicates the distance range, and the vertical axis indicates the cumulative number of point events. As shown in Figure 4a, the observed blue curve is above the upper envelope of the curves under the CSR hypothesis and, hence, we can reject the CSR hypothesis with a 0.95 confidence level [37]. That is, hospitals tend to cluster in that distance range, indicating that the distribution of hospitals is not even in main urban districts.

When the downtown area is used as the study scope instead of the main urban districts, the results of the network auto K-function method of hospitals are quite different. In Figure 4b, the observed curve is between the upper and lower envelope curves and, thus, we can reject the CSR hypothesis with 95% confidence in this range. That is, hospitals tend to be in a random distribution in the downtown area. This indicates that there is an apparent imbalance of hospital development between the main urban districts and the downtown area in Nanjing.

**Figure 5:**
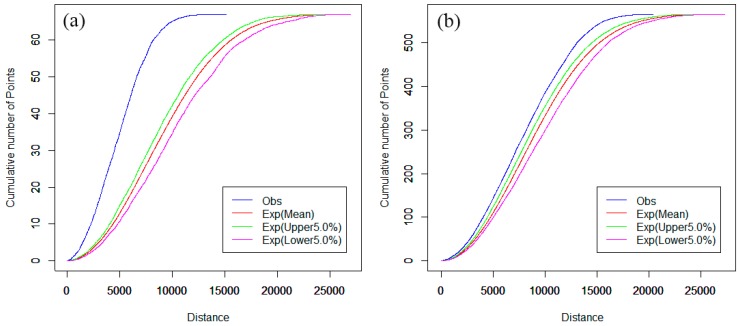
(**a**) Network auto K-function analysis of first- and second-class hospitals and (**b**) network auto K-function analysis of other hospitals in main urban districts.

For the spatial cluster pattern analysis of the combined first- and second-class hospitals, Figure 5a shows that the observed curve of the network K-function is obviously higher above the upper envelope curve under the CSR hypothesis. However, the observed curve is slightly above the upper envelope curve for other types of hospitals, as shown in Figure 5b. In other words, the network aggregation of the first- and second-class hospitals is much stronger than the network aggregation of other hospitals in the main urban districts. This may be because the majority of first- and second-class hospitals were built long ago and are mainly located in the downtown area. The planners did not consider future expansion of the city and population growth. Although probably reasonable in the past, spatial distributions do not seem to be reasonable at present. Other categories of hospitals, such as private hospitals, clinics, and outpatient hospitals, were built after China’s reform and opening up, when the government and planners began to realize that, for fairness and accessibility, these hospitals should be planned in detail.

**Figure 6:**
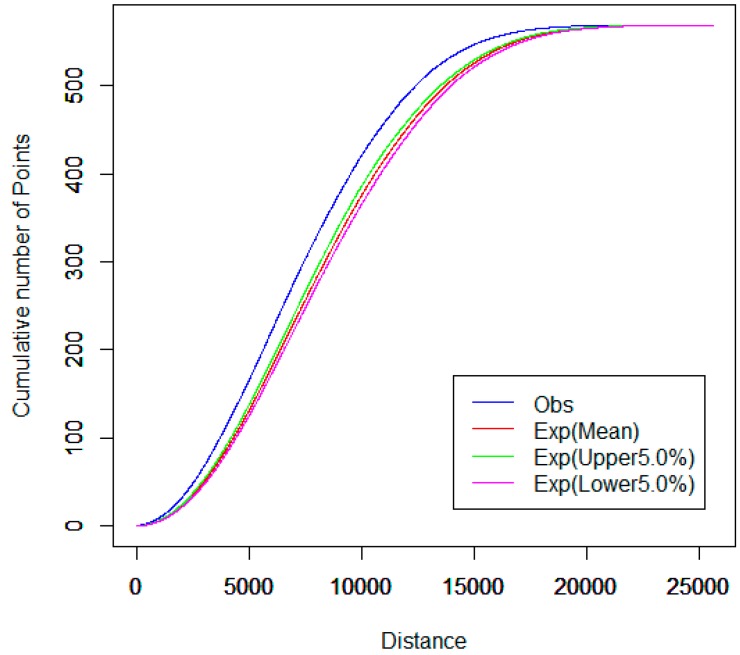
Network cross K-function analysis between hospitals and pharmacy stores in main urban districts.

Furthermore, we investigated the effects of hospital points on the spatial distribution of pharmacy stores in the street network space. The network cross K-function analysis results between pharmacy stores and hospitals are shown in Figure 6. Since the observed curve is slightly above the upper envelope curve, the pharmacy stores in this range tend to cluster around hospitals along the road network. This is consistent with the fact that drugs in hospitals are so expensive that many patients prefer to buy drugs from nearby pharmacy stores in China. However, reducing hospital drug prices has become an imperative task for the current reform of Chinese healthcare in 2016, which will have an important impact on the location selection of new pharmacy stores.

### 4.3. Street Centrality Indexes and Correlation Analysis

**Figure 7:**
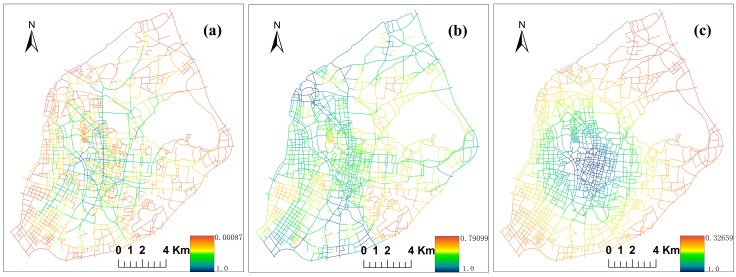
(**a**) Street betweenness; (**b**) straightness; and (**c**) closeness centrality in main urban areas.

**Figure 8:**
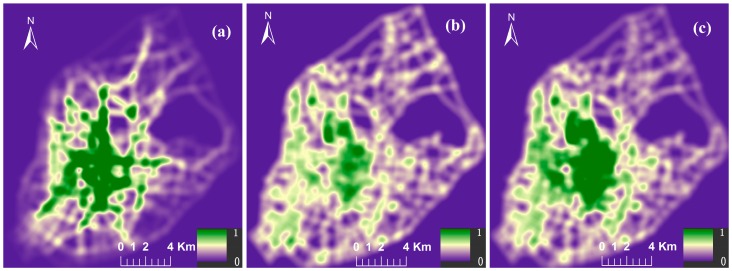
(**a**) KDE of street betweenness; (**b**) straightness; and (**c**) closeness centrality in main urban areas.

To analyze the relationship between hospitals and street centralities, the planer KDE values of street centrality and hospitals NetKDE should be computed and converted to the same raster framework. The eight categories of weighted hospitals NetKDE should be computed using the same calculation method as that used in Section 4.1. Then, the planar KDE values of these hospitals’ NetKDE results were generated (not shown). The geography of three street centrality indexes ($C^{B},C^{S},C^{C}$) in main urban districts was calculated using the Urban Network Analysis tool [38], as shown in Figure 7. Figure 8a–c show the planar KDE of Figure 7a–c, respectively. The bandwidth and cell size were set to 600 m and 20 m when computing planar KDE, which is the same as computing NetKDE.

Spearman’s *r* between different categories of hospitals and three street centrality indexes were computed in the same raster framework. Table 2 is the Spearman’s *r* correlation results between eight categories of hospitals and three street centralities, calculated using SPSS 19.0 software (IBM, Chicago, IL, USA). Note that all correlation coefficients shown in the following analysis were significant at the 0.01 level. Table 2 shows that all eight paired correlation values have positive relationships, which are all higher than 0.45. However, the distribution of the street centralities correlates marginally with first-class hospitals’ activities, and also with second-class hospitals’ activities, although to a slightly higher extent. From the results of the abovementioned hot spots and spatial clusters, this may be because the most bed resources are excessively concentrated in the downtown area, and first- and second-class hospitals are mainly concentrated in these areas. By contrast, third-class hospitals, outpatient facilities, and clinics have higher correlations with the street centralities among the five hospitals in the first group. Clinics have the highest correlation values with street centralities. Furthermore, private hospitals have higher correlation values with street centralities compared to public hospitals. Small size and smart flexibility may make private hospitals meet market needs more easily. In addition, these types of hospitals were established later, when more spatial ideas about equity and competitiveness were integrated in the location selection of new hospitals. All of the hospitals have by far the strongest correlation with the three street centralities whose *r* values are above 0.8. The results indicate that hospitals with high weights in Nanjing tend to concentrate in the areas with better centralities.

Betweenness correlates with a relatively higher *r* value compared to straightness and closeness, which is consistent with previous research [19]. Betweenness refers to the potential of one road traversed by passengers to be between other road nodes. Although the hospitals are not located in an origin or destination, the hospital may take advantage of its pass-through nexus location to attract patients. Hence, a high value of betweenness centrality often implies a high concentration of hospital facilities.

## 5. Conclusions

This study analyzed the spatial distribution characteristics of healthcare facilities in Nanjing by using point pattern analysis and the correlation analysis method. There are two major contributions of this study. First, it analyzed the influence of weighting point events on the analysis results in order to attach importance to the network-constrained point pattern analysis. Second, it combined the network point pattern method and correlation analysis to study the distribution pattern of healthcare facilities comprehensively and to analyze the basic principle of their locational choice. All of these results can be extended to examine the reasonability of the existing service facilities distribution and to optimize the location selection of new service facilities.

However, several problems remain for further study. First, the location of healthcare facilities is influenced by the income of urban inhabitants, hospital fees, population distribution, and other factors. More comprehensive parameters should be considered to investigate the reasonability of location selection of healthcare facilities in future studies. Second, a spatial accessibility model (e.g., gravity model and two-step floating catchment area) should be combined with the methods proposed in this study for further analysis of the interaction between demand and supply location [39]. Third, roads can be categorized to analyze their attraction to different categories of healthcare facilities. As we know, roads with a higher level have greater traffic volume, which could attract more allocation of hospitals. The two non-spatial characteristics of roads and facilities will be considered together in point pattern analysis for more comprehensive and detailed results.

## Figures and Tables

**Table 1:** Numbers of hospitals divided by comprehensive strength and ownership in main urban districts.

Comprehensive Strength	Numbers	Ownership	Numbers
First class	33	Private	376
Second class	38	Public	356
Third class	152		
Fourth class	135		
Fifth class	374		
Total number	732		732

**Table 2:** Correlation values between street centralities and different categories of hospitals.

Categories	*C^S^*	*C^C^*	*C^B^*
First class	0.493	0.523	0.529
Second class	0.509	0.526	0.527
Third class	0.729	0.748	0.748
Fourth class	0.657	0.679	0.698
Fifth class	0.786	0.813	0.824
Public	0.777	0.799	0.812
Private	0.811	0.832	0.840
All hospitals	0.846	0.866	0.870
